# HDAC4 Inhibitors as Antivascular Senescence Therapeutics

**DOI:** 10.1155/2022/3087916

**Published:** 2022-06-29

**Authors:** Chuoji Huang, Zhongxiao Lin, Xiaoyan Liu, Qian Ding, Jianghong Cai, Zhongyi Zhang, Peter Rose, Yi Zhun Zhu

**Affiliations:** ^1^State Key Laboratory of Quality Research in Chinese Medicine and School of Pharmacy, Macau University of Science and Technology, Macau 999078, China; ^2^Shanghai Key Laboratory of Bioactive Small Molecules, Department of Pharmacology, School of Pharmacy, Fudan University, Shanghai 201203, China; ^3^School of Biosciences, University of Nottingham, Loughborough, Leicestershire, UK

## Abstract

Aging is an inevitable consequence of life, and during this process, the epigenetic landscape changes and reactive oxygen species (ROS) accumulation increases. Inevitably, these changes are common in many age-related diseases, including neurodegeneration, hypertension, and cardiovascular diseases. In the current research, histone deacetylation 4 (HDAC4) was studied as a potential therapeutic target in vascular senescence. HDAC4 is a specific class II histone deacetylation protein that participates in epigenetic modifications and deacetylation of heat shock proteins and various transcription factors. There is increasing evidence to support that HDAC4 is a potential therapeutic target, and developments in the synthesis and testing of HDAC4 inhibitors are now gaining interest from academia and the pharmaceutical industry.

## 1. Introduction

Despite gains in average life expectancy, the aging process poses many challenges in the management of age-related diseases such as neurodegeneration and cardiovascular diseases (CVDs). Neurodegenerative diseases have become the most debilitating maladies in older people with risk increasing with advancing age [1]. Neurodegeneration has both hereditary and biochemical traits resulting in progressive degeneration of neurons [2]. Similarly, CVDs are responsible for approximately 4 million deaths each year in China and 17.9 million worldwide [3]. CVDs are caused by multiple factors including epigenetic modification and reactive oxygen species (ROS) [4–6]. Elevations in ROS are widely associated with aging and diseases being produced by four systems including NOX xanthine oxidases, myeloperoxidase, and nitric oxide synthases (NOS) [7–10]. Among these, NOX and NOS are associated with age-related diseases, DNA damage, and mitochondrion dysfunction, therefore influencing epigenetic change.

Genetic inheritance plays an important role in longevity and in age-related diseases [1]. Oxidation or histone acetylation results in altered protein homeostasis, DNA damage, and epigenetic changes, occurring in aged tissues [11]. Histone acetyltransferases (HATs) and histone deacetylases (HDACs) are two classes of enzymes regulating histone acetylation and deacetylation. Deacetylation of HDACs results in positive charges in the condensation of chromatin and thereby turns off gene transcription [12]. Among the HDAC superfamilies, HDAC4 is of interest since this enzyme is located both in the nucleus and cytoplasm and may act on more than simply cellular histones. This factor alone suggests that HDAC4 could have the potential in the treatment of neurodegeneration or CVDs.

### 1.1. The Subcellular Location and Substrates of HDAC4

In mammals, there are 18 types of HDACs recognized, and these are divided into classes I, II, III, and IV based on structure and homology with yeast HDACs. Class I types consist of HDACs 1, 2, 3, and 8, which are expressed and located in the nucleus [13]. Class III is composed of a family of sirtuins, and their activation is dependent on NAD^+^. HDAC11 is the only member of class IV and having structural similarities to class I HDACs. Class II proteins can be further divided into two subgroups: class IIa (HDACs 4, 5, 7, and 9) and class IIb consisting of HDACs 6 and 10. Interestingly, only class II HDACs exhibit tissue-specific patterns of expression. For example, HDAC4, which will be mainly discussed in this paper, is highly expressed in the brain, heart, and skeletal muscle [14].

**Figure 1:**
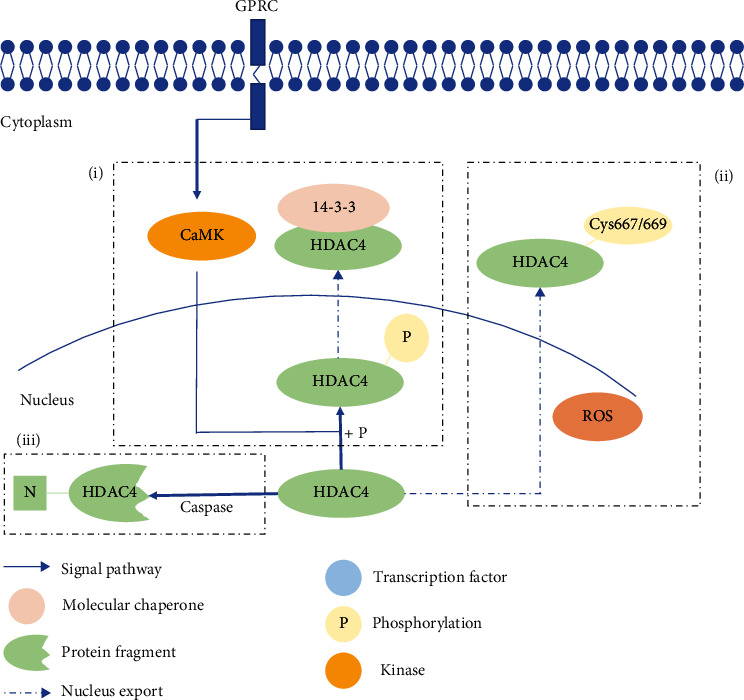
The scheme of HDAC4 partitioning between the nucleus and cytoplasm. (a) HDAC4 is phosphorylated by a kinase like CaMK and then combines 14-3-3, inducing nucleus export. (b) Oxidation of Cys667/669 of HDAC4 also promotes nuclear export. (c) The cleavage of HDAC4 causes N-term retention in the nucleus, and the N-terminal sequence has functional activity.

In contrast to class I HDACs, HDAC4 shuttles between the nucleus and cytoplasm. The location of HDAC4 plays an important function in dictating the physiology and pathological role of this protein within cells and tissues. HDAC4 has the tendency to be maintained in the cytoplasm in neurons and in the nucleus of myoblasts. The key to the subcellular location of HDAC4 is its phosphorylation status. Phosphorylated HDAC4 binds to the chaperone protein 14-3-3, inducing nuclear export [15]. Phosphorylation of HDAC4 can be mediated by a diverse array of kinases, namely, calcium/calmodulin-dependent protein kinase (CaMK) [16], extracellular signal-regulated kinases 1 and 2 (ERK1/2) [16], protein kinase A (PKA), and glycogen synthase kinase 3 (GSK3) [17]. CaMK play a significant role in the maintenance of neurons and in the export of HDAC4 driven by calcium influx induced by synaptic activity in neurons [18]. Serine residues at positions 210, 246, 350, 467, and 632 are key phosphorylation sites in this protein [16, 19]. In addition, ROS can also induce nuclear export. In this instance, NOX4 produces H_2_O_2_ which directly oxidizes cysteine residues 274/276 in DnaJb5 and cysteine-667/669 in HDAC4, to promote nuclear targeting of HDAC4 export [20, 21]. Interestingly, proteolytic cleavage can also influence the location of HDAC4 in cells. Following cleavage by caspases, HDAC4 leaves the nuclear localization signal containing fragments accumulated in the nucleus [22, 23]. The fragments lose the C-terminal catalytic domain but retain the combination with MEF2C. Moreover, the fragments show an increased repressive effect on Runx2- and SRF-dependent transcription (Figure 1) [24].

**Figure 2:**
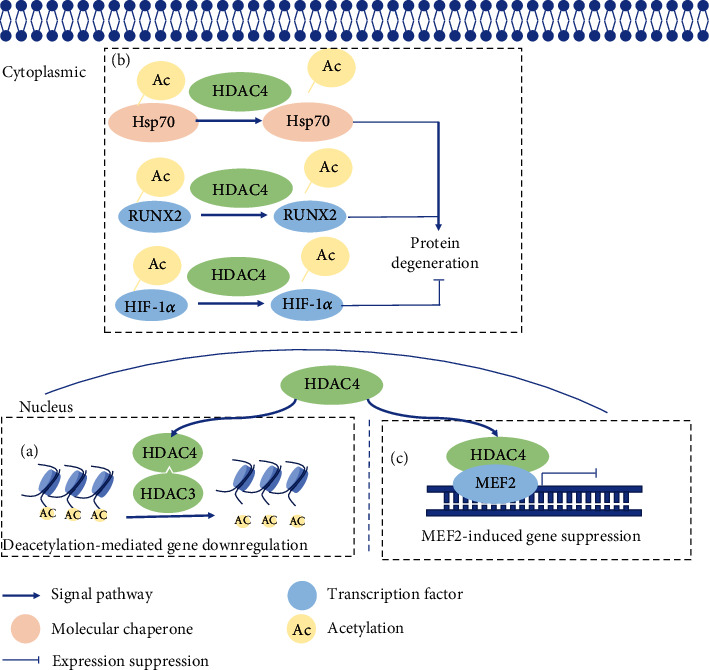
A schematic of HDAC4 function and interaction with cellular substrates. (a) HDAC4 deacetylates histones H3 and H4, tightening the linkage between DNA and nucleosomes and turning off gene transcription; (b) HDAC4 deacetylates nonhistone proteins like Hsp70 and transcription factors therefore altering protein degeneration; (c) HDAC4 binds to MEF2 and then suppresses gene transcription, playing a vital role in muscle-related development and diseases.

Consistent with its cellular location, HDAC4 is involved in removing acetyl groups from both histones and nonhistone proteins with a zinc-containing catalytic domain. Unlike class I HDACs, HDAC4 only gains deacetylase activity only when interacting with HDAC3 and RbAp48 [15]. The catalytic domain tends to form a multiprotein functional complex. Deacetylation of histone H3 and histone H4 suppresses gene expression. In addition, heat shock protein 70 (Hsp70) can be acetylated at lysine 77. Hsp70 is acetylated by ARD1 in the early cellular stress response and deacetylated by HDAC4 in the late. Deacetylated Hsp70 contributes to protein degradation [25]. Transcription factors are another kind of substrates deacetylated by HDAC4. Runx-2 and HIF-1*α* are known transcription factors being deacetylated by HDAC4. Acetylated Runx-2 inhibits Smurf1-mediated degradation, but deacetylated HIF-1*α* shows increased stability (Figure 2) [26–29].

In addition to its catalytic function, HDAC4 can directly interact with other cellular proteins. For example, HDAC4 directly binds to and represses MEF2-mediated expression of GATA4 and Nkx2-5. As a result, HDAC4 prevents myogenesis. The repression of gene transcription by the MEF-2/HDAC complexes is suppressed due to CaMK-induced translocation of HDAC4 and HDAC5 to the cytoplasm.

## 2. HDAC4 Promotes Age-Related Diseases

Numerous studies show that HDAC4 has a broad interaction with different kinds of proteins and is involved in several physiological pathways such as myogenesis and oxidative stress. This sensitive balance means that HDAC4 plays important roles in growth and development. However, dysfunction of HDAC4, which often occurs during aging, may precipitate conditions like hypertension, cardiovascular diseases (CVDs), and neurodegeneration.

Vascular calcification is the pathological accumulation of calcium phosphate crystals in the medial and intimal layers of vascular walls and is tightly linked with metabolic diseases such as chronic kidney disease, diabetes, and vascular diseases viz. atherosclerosis [30]. Pathologically, there are two major forms of vascular calcification with both existing in the same clinical condition. The first type is intimal calcification associated with atherosclerosis-linked lipid and cholesterol accumulation under the injured endothelium. The second type is medial calcification, also known as Mönckeberg's sclerosis, which involves the deposition of minerals within the vascular smooth muscle layers [31]. Ting and colleagues revealed novel findings on the involvement of HDACs and their modifiers in the development of vascular calcification. In human aortic smooth muscle cells, inhibition of HDAC mitigates the effect of Notch protein to increase smooth muscle *α*-actin levels, indicating that HDAC activity is required for Notch signaling during differentiation [32]. HDAC4 and HDAC5 are regulated in a CaMKII-dependent manner in vascular smooth muscle cells [33, 34]. Inhibition of HDAC using butyrate abrogates the activation of Akt. This results in differential effects on the downstream targets of Akt, promoting signaling cross-talk and resulting in vascular smooth muscle growth through proliferation arrest [35]. RUNX2 is one of the downstream targets of Akt signaling via hydrogen peroxide activation and has an increased expression level in vascular calcification [36]. HIF-1*α* promotes the calcification and osteogenesis of vascular smooth muscle cells to build extracellular matrix calcification. HIF-1*α* is downregulated by ROS scavengers and HDAC4 inhibitors [27, 37, 38]. Also, HIF-1*α* is reported to be a key transcription factor in mitochondrial dysfunction in hypoxia response [39]. These results show that ROS and HDAC4 have a synergistic effect on vascular aging and calcification. Researchers at Yale University have shown that HDAC activity is associated with hypertension by increasing MEF2 activity in endothelial cells following treatment with a class IIa histone deacetylase inhibitor [34]. This innovation may be valuable as a treatment solution for pulmonary hypertension, by offering a means of restoring MEF2 activity using class IIa histone deacetylase inhibitors. Therefore, the critical function of HDACs in vascular biology could be exploited to employ HDACs as a molecular target for treating hypertension [40].

**Figure 3:**
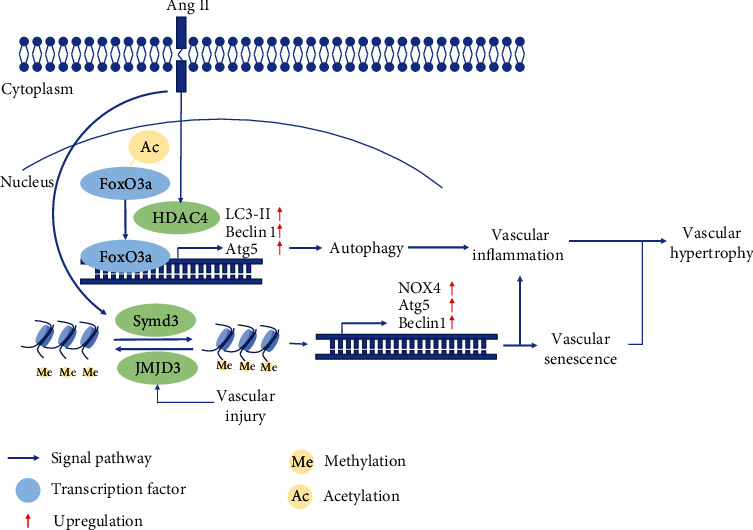
Epigenetic modification in vascular diseases. Ang II induces the expression of HDAC4 and Symd3. The former deacetylates FoxO3a, upregulating the levels of LC3 II, Atg5, and Beclin 1. The latter methylates H3K4 inducing NOX4, Atg5, and Beclin 1 expression. HDAC4 and Symd3 both mediate vascular inflammation and senescence. JMJD3 demethylates H3K27 and induces NOX4, Atg5, and Beclin 1 expression, resulting in the pathological features of vascular senescence and inflammation. All of these may lead to vascular hypertrophy.

CVD is highly prevalent and is the leading cause of mortality and morbidity in developed countries. CVD refers to a broad spectrum of diseases affecting the cardiovascular system and includes the heart and blood vessels. One common condition is atherosclerosis, a progressive disease in which the inner layers of the artery walls become thick and irregular because of the deposition of fat, cholesterol, and other substances. Interestingly, HDAC4 plays a vital role in mediating cardiovascular diseases since (1) Ca^2+^/calmodulin-dependent protein kinase- (CaMK-) II promotes hypertrophic growth via phosphorylation of HDAC4 in cultured cardiomyocytes, (2) activation of HDAC4 promotes angiotensin II-induced vascular smooth muscle hypertrophy, and (3) CaMKII-mediated cardiac hypertrophy can be altered by interfering with the HDAC4-MEF-2 signaling pathway [41]. This link is likely due to HDAC4's ability to promote reactive oxygen species- (ROS-) dependent vascular inflammation and the development of hypertension in spontaneously hypertensive rats [33]. Similarly, in a mouse model of vascular inflammation, Ang II-induced production of proinflammatory mediators, such as IL-6, VCAM-1, COX2, and iNOS, is attenuated by knockdown of HDAC4. HDAC4 is activated by Ang II and deacetylates transcription factor FoxO3a, inducing upregulation of LC3-II, Beclin 1, and Atg5. In addition, to determine whether HDAC4 mediates the inflammatory response, Tasquinimod (Taq), an inhibitor of HDAC4, was tested [42]. The levels of proinflammatory mediators decrease significantly in rat primary endothelial cells cotreated with Ang II/Taq but increase in Ang II-treated cells. Immunofluorescence further confirmed that treatment with siRNA HDAC4 or Taq decreases the expression of HDAC4 and VCAM-1 proteins. The Ang II-induced inflammatory response is alleviated by the inhibition of HDAC4 (see Figure 3). In addition, ROS are critical components of Ang II function [43]. Among the various forms of ROS, superoxide anion (^·^O2^−^), hydrogen peroxide (H_2_O_2_), nitric oxide (NO), and peroxynitrite (ONOO^−^) are particularly important in the cardiovascular system [44]. In the cardiovascular system, NOX is the main producer of vascular ROS, and NOX4 is induced by Ang II [45]. Interestingly, NOX4-produced H_2_O_2_ also influences the location of HDAC4.

Vascular inflammation is significantly correlated with hypertension and CVD. Vascular injury or damage accumulated in vascular senescence also results in vascular endothelial cell proliferation and migration, causing hypertrophic growth [46]. Vascular inflammation is observed in the two situations [47]. In the first, Smyd3 (SET and MYND domain-containing protein 3), a methyltransferase inducing trimethylation of lysine 4 on histone 3 (H3K4me3), participates in Ang II-induced vascular senescence. Knockout of Smyd3 in mice can significantly alleviate Ang II-induced vascular senescence. Proinflammatory mediators such as VCAM-1 and iNOS are upregulated in Ang II-induced WT mice but blocked in Smyd3^−/−^ mice [48, 49]. In a vascular injury model, JMJD3 (Jumonji domain-containing protein 3), a histone demethylase inducing demethylation of lysine 27 on histone 3 (H3K27), induces increased expression of NOX4, Atg5, Beclin 1, and iNOS, respectively. Changes in protein expression levels indicate oxidative stress and vascular inflammation (Figure 3) [50, 51].

**Figure 4:**
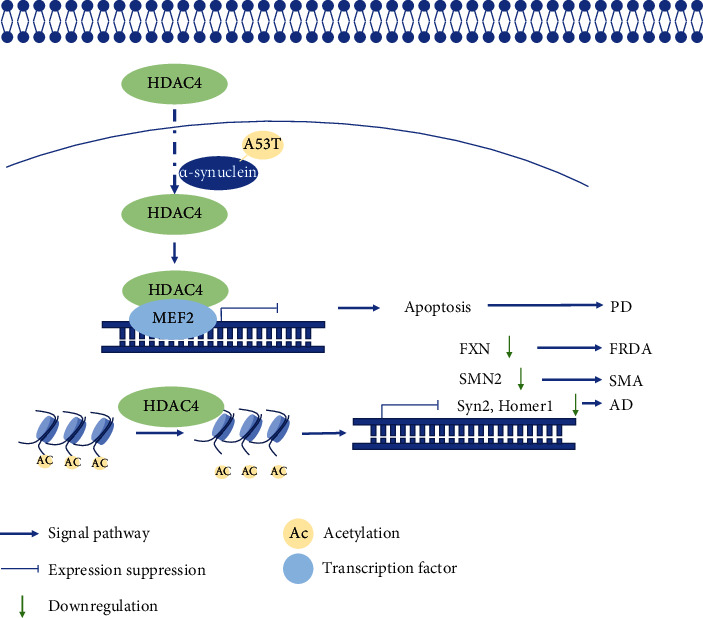
HDAC4 mediated neurodegeneration. HDAC4 deacetylates histones, subsequently tightens the linkage between nucleosomes and DNA, and represses the levels of FXN, SMN2, Syn2, and Homer1. These gene expression changes mediate FRDA, SMA, and AD. A53T mutant *α*-synuclein accumulates HDAC4 transport into the nucleus, which binds to MEF2 mediating neuron apoptosis and leading to PD.

Ang II-induced vascular injury indicates a crucial role of immune cells in disease progression. The activated macrophages and T cells regulate vascular inflammation driving vascular injury. Macrophage colony-stimulating factor (m-CSF) deficiency reduces the number of vascular macrophages in Ang II-induced endothelial dysfunction, vascular inflammation, and hypertension [52]. The evaluated expression of SMYD3 promotes the activation of ALOX-15, which acts as a marker of anti-inflammatory macrophages [53]. Meanwhile, JMJD3 is also involved in the profibrotic signature of macrophage-derived foam cells via RNA sequencing [54]. In addition, *γδ* T cells mediate Ang II--induced vascular injury. Comparison of TCR*δ*^−/−^ between WT mice showed the CD4^+^ CD69^+^ and CD4^+^ CD69^+^ T cells activated in WT mice and blunting in TCR*δ*^−/−^ mice [55]. Vascular inflammation is also associated with many forms of neurodegenerations, including Alzheimer's disease, Parkinson's disease, and amyotrophic lateral sclerosis [56]. Alzheimer's disease (AD) is the most common form of dementia, a brain degenerative disease affecting nearly 10% of the population over 65 years of age [57, 58]. Amyloid-*β* (A*β*) is widely considered a key contributor to the pathophysiology of AD and induces brain inflammation. Inflammation in endothelial cells is characterized by the expression of VCAM-1 and ICAM-1. The elevated expression level of NLRP3 has been observed in human brain and model mice, activating the production of proinflammatory cytokines like IL-1*β*, IL-18, and gasdermin D [59, 60]. In addition, the elevated expression level of HDAC4 was confirmed in the brain of AD patients and in mouse models. Indeed, the oral administration of Taq increases the levels of Syn2 and Homer1, which are upregulated in wide mice compared to 3xTg-AD mice [61–64] (Figure 4).

In other neurodegenerative conditions like Parkinson's disease (PD), HDAC4 may also be important. PD is the second most common neurodegenerative disorder affecting approximately 0.2% of the global population and 1% of people aged over 60 [65]. In a recent case-control study involving 33 patients and 27 healthy subjects, it was shown that high expression levels of VCAM-1 and angiogenic microRNAs were linked to vascular inflammation [66]. The A53T mutant *α*-synuclein induces nuclear accumulation of HDAC4, promoting neuronal apoptosis through suppressing the activity of MEF2 [67] (Figure 4). Though there is no HDAC4 inhibitor for therapeutic purposes, the nuclear accumulation of HDAC4 discloses avenues for possible intervention using therapeutics.

In addition, other conditions like Friedreich's ataxia (FRDA), spinal muscular atrophy (SMA), and amyotrophic lateral sclerosis (ALS) are also vascular inflammation-associated neurodegenerations [68, 69]. HDAC inhibitors increase FXN expression by ~15% in FRDA and ameliorate the disease phenotype in animal models [70–73]. The severity of SMA is inversely correlated with the relative amount of SMN protein. Several inhibitors including butyrate, valproate, phenyl-butyrate, and vorinostat, class I and II HDAC inhibitors, are effective in upregulating the expression of SMN2 in fibroblasts obtained from patients suffering from SMA, which is associated with improved survival, weight loss, and motor behavior [74–78]. In ALS research, two studies revealed that the pan-HDAC inhibitor TSA or sodium phenylbutyrate ameliorates axonal degeneration leading to motoneuron-related death and enhancing the motor functions in the SOD1^G93A^ mouse model [79, 80] (Figure 4).

## 3. The Research and Development of HDAC4 Inhibitors

HDAC4 inhibitors have been proved effective for cancer, CVDs, and neurodegeneration. The synthesis of HDAC4 inhibitors has been a subject of clinical research for several decades. The specificity of inhibitors has also developed over time from generic pan-HDAC inhibitors to the more refined class-specific inhibitors.

Trichostatin A (TSA) is an archetypal classical HDAC inhibitor, which has been used widely by researchers. TSA is a pan-HDAC inhibitor, which inhibits both class I and class II HDACs. TSA was originally reported as a fungistatic antibiotic obtained from culture broths of *Streptomyces platensis*. Other inhibitors have also been reported and include vorinostat. This compound is also known as suberoylanilide hydroxamic acid (SAHA) and was designed and optimized based on similarity to the structure of TSA. Vorinostat has been widely used to treat cutaneous T cell lymphoma. As summarized, the synthesis of SAHA indicates that natural products could be a possible source for the identification and development of highly selective HDAC4 inhibitors.

Other compounds of interest include LMK235 (N-((6-(hydroxyamino)-6-oxohexyl)oxy)-3,5-dimethylbenzamide), a potent hydroxamate-based HDAC inhibitor. LMK235 specifically inhibits HDAC4 and shows equipotent efficacy to HDAC4 as pan-HDAC inhibitors like SAHA [81]. Moreover, two class IIa HDAC inhibitors, TMP195 and TMP269, which both contain a common metal-binding group, have increased specificity [82].

Looking to the future, multifold HDAC4 inhibitors are currently in various development (R&D) pipelines. Of the available research, these molecules are showing promise in the treatment of different types of cancer, autoimmune diseases, peripheral pain, psychiatric disorders, and inflammation. BMN-290, an HDAC4i inhibitor developed by Scripps Research Institute and BioMarin Pharmaceutical Inc., has been assessed for the treatment of neurodegenerative diseases The compound reverses FXN silencing and is in the preclinical stage. Two other HDAC4 inhibitors, mocetinostat dihydrobromide and a class IIa HDAC inhibitor, both developed by MethylGene Inc., are undergoing extensive clinical trials for the treatment of multiple cancers including advanced solid tumor and metastatic non-small-cell lung cancer. The former compound was designed to target HDACs 4, 5, and 7, while the latter was designed as HDAC 1, 2, 3, 4, 7, and 11 inhibitors. In addition, the novel HDAC4 selective inhibitor, CHDI-00381817, is being investigated by the Cure Huntington's Disease Initiative (CHDI) foundation for its potential use in treating Huntington's disease (HD) (Table 1). These four HDAC4 inhibitors are more selective than TSA and SAHA, but some of these drugs could be further developed to enhance selectivity.

Another four HDAC4 inhibitors are under preclinical stage evaluation. SIK3 is a checkpoint inhibitor, is developed by iOmx Therapeutics AG, and is designed as a HDAC4 inhibitor. Similarly, KYAN-001, developed by Georgetown University and Kyan Therapeutics Inc., is designed as a HDAC4- and HDAC6-specific inhibitor. These two molecules are both aimed at cancer therapy. KRA-1641 is developed by Karus Therapeutics Ltd., and a histone deacetylase-4 inhibitor (oral, neurodegenerative diseases/amyotrophic lateral sclerosis) is developed by Acetylon Pharmaceuticals Inc. The two are HDAC4-specific inhibitors.

### 3.1. Our Work and Hypothesis

**Figure 5:**
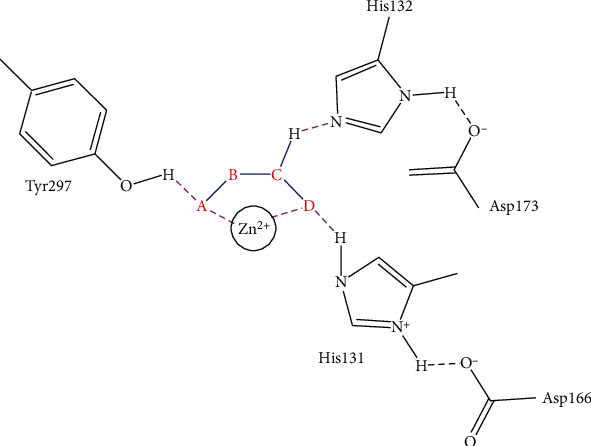
The structures of HDAC are divided into groups A, B, C, and D for the attachment or binding of histone deacetylases (HDACs). Group A inhibitors are soft, with nonbonding electron-pair donors that coordinate the zinc ion and H-bond acceptor to accept a hydrogen bond from tyrosine –OH. An H-bond could be donated to the phenol oxygen atom of Tyr297. Group B links the zinc-chelating moiety to the spacer and hence is at least trivalent. Group C includes H-bond donors to residue His132; consequently, they are trivalent or of higher valency. Group D includes proton donors that protonate His131, subsequently accepting an ionic H-bond from it and forming a strong interaction with the zinc ion.

In a recent study, we collected the chemical structures of several HDAC4 inhibitors from patent documents and tried to evaluate their characteristics related to clinical use. Based on the information, we attempted to design several novel selective HDAC4 inhibitors. The structures of these HDAC4 inhibitors can be divided into four groups: A, B, C, and D, as shown in Figure 5 [79, 83]. Small molecules with structures similar to HDAC4 were considered possible HDAC4 inhibitors. Different functional groups including OCH_3_, OH, N(CH_3_)_2_, NO_2_, and small alkyl chains were added to the compounds to increase their affinity for HDAC4. Initially, these molecules were screened using the molecular operating environment software. The docking results obtained from the software predicted that these small molecules would inhibit HDAC4, which remains to be further confirmed by *in vitro* and *in vivo* studies.

## 4. Discussion

Here, we report on the role of HDAC4 and its utility as a potential therapeutic target in the treatment of age-related diseases. Histone deacetylation, nonhistone deacetylation, and protein complexes are induced by HDAC4 and participate in aging and neurodegeneration. HDAC4 is regulated by variable intercellular features including abundance, posttranscriptional regulation, and location. ROS determines HDAC4 location and accumulates in the nucleus/cytoplasm in aging and neurodegeneration. ROS induces DNA damage, apoptosis, and protein degradation, all of which cause DNA structural variation. In addition, the level of ROS is influenced by HDAC inhibitors. These mechanisms indicate associations between ROS and HDAC4.

Hypertension, CVD, and neurodegenerations are reported to be related to inflammation. Indeed, vascular inflammation is largely driven via injury and aging processes. Vascular injury induced by stroke is linked to vascular inflammation and induced PD. However, the linkage between damage accumulation along with aging and disease has not been fully explored. What is known is that the epigenetic landscape and oxidative stress are intimately linked and likely play a role in age-related diseases. It is likely that HDAC4 is important in these processes. Indeed, the activity and location of HDAC4 both act on age-related diseases. The activity of HDAC4 relates to muscle disease, and the location preferably relates to neurodegeneration. Hence, HDAC4 inhibitors may show promise as potential therapeutics.

HDAC4 inhibitors have been recognized as a potential therapeutic approach for several diseases. Small molecules with similar structures to existing HDAC4 inhibitors could be considered possible new HDAC4 inhibitors. For example, SAHA has a similar structure to TSA. Derivatives of hydroxamate and trifluoromethyl oxadiazole should also be considered potential inhibitors. However, most HDAC4 inhibitor research and associated clinical trials largely focus on cancer treatment. Therefore, in the future, it may be timely to assess HDAC4 inhibitors in neurodegenerative conditions. Since the therapeutic drug has already been developed, potential use in neurodegenerations could be evaluated [84]. Our group also designed and synthesized novel HDAC4 inhibitors from natural products, and the work will be published soon.

The dysfunction of HDAC4 causes diseases, but normal HDAC4 plays a critical role in differentiation and development. HDAC4 is required for learning, memory, and synaptic plasticity [85]. Although the research for HDAC4 has proceeded for over 20 years, there are still lots of research gaps remaining. The lack of HDAC4-specific inhibitors and the abundant posttranscriptional regulation make the verification a complicated work. Further studies will elucidate the mechanisms of HDAC4 and provide more feasible drug design groups.

## Figures and Tables

**Table 1:** HDAC4 inhibitors in the research and development pipelines.

Drug ID	Representative structure(s)	IC50
LMK-235	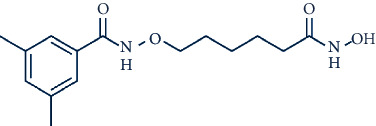	11.9 and 4.2 nM against HDAC4 and HDAC5
TMP-195	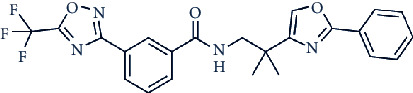	59, 60, 26, and 15 nM against HDACs 4, 5, 7, and 9
TMP-269	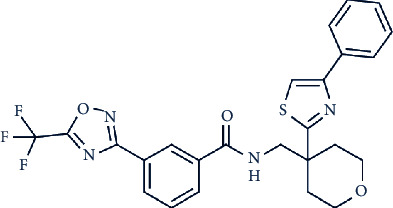	157, 97, 43, and 23 nM against HDACs 4, 5, 7, and 9
BMN-290	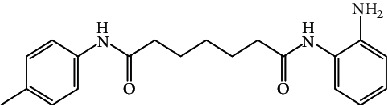	NA
Mocetinostat dihydrobromide (MG-0103)	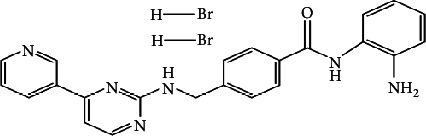	0.05, 0.2, 1, and 20 *μ*M against HDACs 1, 2, 4, 6, and 8, respectively
Class IIa HDAC inhibitors, MethylGene	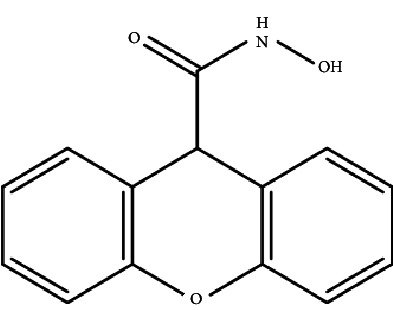	0.25, 0.11, and 0.05 *μ*M against HDACs 4, 5, and 7, respectively
CHDI-00381817	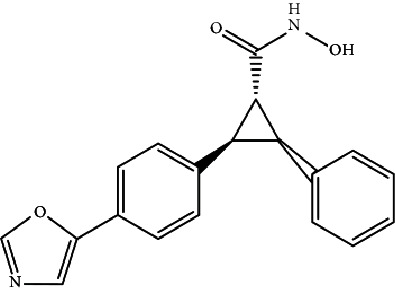	0.02 *μ*M against HDAC4
